# Forecasting the Monkeypox Outbreak Using Limited Data: A Case Study of Thailand

**DOI:** 10.1155/ipid/7199833

**Published:** 2025-11-24

**Authors:** Sherif Eneye Shuaib, Jirapond Muangprathub, Arthit Intarasit, Pakwan Riyapan

**Affiliations:** ^1^Department of Mathematics and Computer Science, Faculty of Science and Technology, Prince of Songkla University, Pattani Campus, Mueang Pattani, Pattani 94000, Thailand; ^2^Faculty of Science and Industrial Technology, Prince of Songkla University, Surat Thani Campus, Surat Thani 84000, Thailand

**Keywords:** exponential smoothing model, forecasting, monkeypox, prophet model, stacking model

## Abstract

**Introduction:**

On August 14, 2024, the World Health Organization (WHO) declared monkeypox (mpox) a Public Health Emergency of International Concern (PHEIC). Shortly thereafter, Thailand reported Asia's first confirmed case of the Clade Ib strain. Given Thailand's high international travel volume, anticipating short-term case trajectories is essential for situational awareness.

**Methods:**

We forecast monthly mpox cases using the Department of Disease Control (DDC) data from July 2022 to September 2024 and evaluate Poisson and negative binomial generalized linear models (GLMs), Holt–Winters exponential smoothing, NeuralProphet, and a stacked ensemble. Rolling-origin cross-validation was used to assess out-of-sample accuracy (MAE, RMSE, and MAPE) and empirical interval coverage (80% and 95%).

**Results:**

Holt–Winters provided the most accurate single-model forecasts by RMSE, while the stacked ensemble yielded the most reliable uncertainty calibration. Forecasts for October–December 2024 suggest relatively low case counts with non-negligible uncertainty, indicating the need for continued vigilance.

**Discussion:**

These findings are exploratory decision-support inputs that complement ongoing surveillance and may help inform adaptive planning under both low- and high-incidence scenarios.

## 1. Introduction

In the 21st century, the world has witnessed several severe viral epidemics and pandemics, including dengue, HIV, MERS-CoV, Swine Flu, Zika, Ebola, Chikungunya, and, most recently, COVID-19 [1]. The COVID-19 pandemic has had a devastating impact on global health and society [2, 3]. Less than 5 years later, the world faces another emerging threat: the rising incidence of mpox. On August 14th, 2024, the World Health Organization (WHO) declared mpox an international public health emergency, its highest level of alert [4].

Mpox is caused by the Monkeypox virus, which belongs to the same family as the variola virus, the causative agent of smallpox. Transmission can occur through both animal-to-human and human-to-human routes, with an incubation period of 6–13 days [4]. Symptoms during the prodrome phase include fever, swollen lymph nodes, headaches, sore throats, and shortness of breath [5]. The virus comprises multiple genetic clades. Clade I has long circulated in Central Africa, often linked to bushmeat consumption [6]. Clade II was responsible for the global 2022 outbreak, largely associated with transmission among men who have sex with men [6]. A newer subclade, Clade Ib, has recently emerged in the Democratic Republic of the Congo, with a higher mortality rate (3%) compared to the 2022 outbreak (0.2%) [7]. Alarmingly, Thailand has reported Asia's first confirmed Clade Ib case [8], prompting neighboring countries to strengthen border screening [9].

Tourism plays a pivotal role in Thailand's economy, attracting millions of international visitors annually. Following the COVID-19 pandemic, tourism receipts exceeded 139 billion Thai baht in December 2023 [10]. Popular attractions, such as temples, festivals, markets, and beaches, often involve close contact, creating potential hotspots for the spread of disease [11]. With Clade Ib now detected in Thailand, there is a pressing need to anticipate potential surges in mpox cases.

Forecasting infectious diseases provides a valuable tool for public health preparedness, enabling authorities to allocate resources more effectively [12, 13]. Traditional statistical models (e.g., GLARMA and ARIMAX) and compartmental models have been widely used in epidemiological forecasting [14–17]. Recent approaches have also incorporated count-based distributions, such as the Poisson and negative binomial, to better capture epidemic dynamics [18, 19]. These models demonstrate that while medical forecasts are inherently uncertain, they can provide valuable situational awareness for policymakers [20].

**Figure 1:**
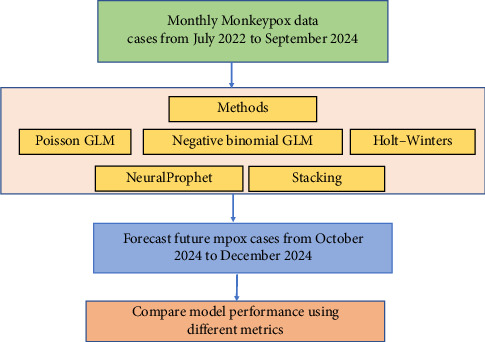
Framework used in this study.

While forecasts cannot replace real-time surveillance, they can provide policymakers with an early indication of possible short-term trends. For Thailand, where mpox Clade Ib has now been detected, such forecasts can support situational awareness during high-risk periods such as peak tourism months. The insights generated in this study should therefore be interpreted as exploratory evidence that complements ongoing surveillance, vaccination, and public health campaigns, rather than as prescriptive recommendations (Figure 1).

The rest of this study is organized as follows.  Section 2 details the dataset used and the methods applied.  Section 3 presents the forecasting results and discussion.  Section 4 concludes the study by summarizing the key findings.

## 2. Methodology

### 2.1. Dataset Description

**Figure 2:**
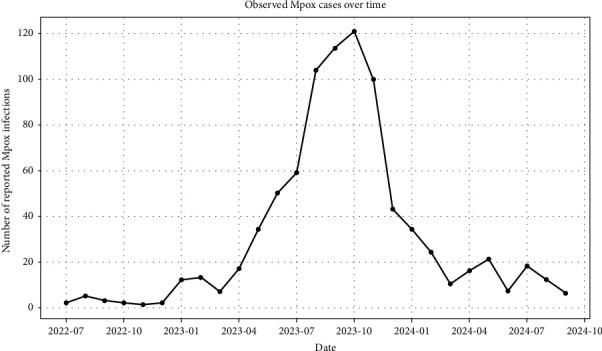
Monkeypox cases recorded in Thailand by the DDC from July 2022 to September 2024.

The dataset used in this study was obtained from the Department of Disease Control (DDC), Ministry of Public Health, Thailand [21], and is provided in the Supporting File (Table S1). Mpox cases are reported on a monthly basis, and the dataset spans July 2022 to September 2024 (as of September 23, 2024).  Figure 2 illustrates the reported monthly mpox counts over this period. Analyses were conducted at the native monthly frequency without interpolation to preserve the integrity of the reporting structure and avoid introducing artificial autocorrelation. This approach ensured that forecasts reflected the true temporal granularity of the surveillance data rather than smoothed or imputed estimates.

Forecasts were generated for the period October–December 2024 using multiple modeling frameworks to capture different temporal and statistical structures in the data. Specifically, we implemented a 3-month moving average (MA) model, Holt–Winters exponential smoothing with an additive trend and no seasonality, NeuralProphet, and two count-based generalized linear models (GLMs): Poisson and negative binomial. The latter two models incorporated a log link to a linear time index to account for potential nonlinearity in temporal dynamics while respecting the discrete nature of case counts. NeuralProphet was trained directly on the monthly series to maintain temporal consistency with the other methods.

To enhance forecast stability and reduce model-specific bias, we constructed a simple stacked ensemble by averaging forecasts from the individual models. Prediction intervals were computed from model residuals, with lower bounds truncated at zero to ensure non-negative case counts. Forecasts and corresponding confidence intervals were generated at a monthly resolution for the last quarter of 2024.

Model evaluation was conducted through rolling-origin (walk-forward) cross-validation with a minimum 12-month training window and one-month-ahead forecasts per origin. This approach mimics real-world forecasting conditions by sequentially updating the training set as new observations become available, thereby capturing temporal dependencies and model adaptability over time. Evaluation metrics included mean absolute error (MAE), root mean squared error (RMSE), mean absolute percentage error (MAPE), and empirical coverage probabilities at 95% and 80% confidence levels. Summary statistics for the dataset are presented in Table 1, while Table 2 reports the out-of-sample performance of all models under the cross-validation scheme.

### 2.2. Forecasting With the MA Method

In many time series applications, a MA method is employed to smooth fluctuations by calculating the mean of the most recent values. The size of the time window plays a vital role in capturing recent trends versus longer-term stability. In this study, we applied a simple MA over the most recent 3 months of data to generate forecasts for October–December 2024. The MA forecast for month *t* + *h* is given by

(1) y^t+h=1n∑i=0n−1yt−i,

where *n* denotes the window size and *y*_*t*−*i*_ represents past observations. This approach provides a simple baseline and, for a fixed window *n*, yields a constant forecast beyond one step [22].

### 2.3. Forecasting With the NeuralProphet

Prophet is an additive regression model developed by Facebook, designed for time-series forecasting [23, 24]. This model is particularly effective in handling complex seasonality, missing values, outliers, and abrupt shifts in trends, making it highly suitable for automatic forecasting tasks. It is supported by a Stan backend, which employs an advanced optimization technique called limited-memory Broyden–Fletcher–Goldfarb–Shannon (L-BFGS) algorithm. For efficient parameter estimation and forecasting, Prophet's forecasting approach is based on a decomposable time-series model described by the following equation:

(2) $$\begin{array}{c} z\left(t\right)=\lambda \left(t\right)+\nu \left(t\right)+\partial \left(t\right)+\gamma \left(t\right). \end{array}$$

In (2), *λ*(*t*) represents the trend component, *ν*(*t*) models seasonal changes, and *∂*(*t*) captures irregularities and other influences. Understanding the trend *λ*(*t*) is critical for accurate forecasting, as it reflects the underlying growth pattern. In this study, a piecewise saturated growth model with a dynamic carrying capacity was utilized to represent the trend, which is defined by

(3) $$\begin{array}{c} \lambda \left(t\right)=\frac{C\left(t\right)}{1+\exp\left(-\left(\kappa +{\alpha \left(t\right)}^{T}\;\varrho \right)\left(t-\left(n+{\alpha \left(t\right)}^{T}\;\chi \right)\right)\right)}. \end{array}$$

In (3), *C*(*t*) denotes the time-varying carrying capacity, *κ* is the growth rate, and *n* is an offset. The growth rate is not constant but varies piecewise, influenced by parameters *α*(*t*),  *χ*, and *ϱ*, which define its structure. NeuralProphet builds on the foundation of the Prophet model, maintaining its strengths while enhancing scalability and accuracy by integrating neural networks with the interpretability of autoregressive (AR) models. This improvement is achieved by utilizing PyTorch and an AR network known as the AR-Net [25]. Instead of relying on traditional least squares to fit a linear model, the AR-Net uses a feedforward neural network to learn the AR forecasting model. This approach enables the AR-Net to be more flexible and effectively handle larger datasets and complex inputs, surpassing the capabilities of linear models. Both Prophet and NeuralProphet excel in handling datasets with significant seasonal patterns.

### 2.4. Forecasting With the Holt–Winters Exponential Smoothing Method

The Holt–Winters exponential smoothing algorithm, introduced in the 1950s through various scientific publications [26–28], is a widely recognized technique for time-series forecasting. It applies exponentially decreasing weights to previous observations, allowing it to capture trends in time-series data. The model is expressed as

(4) r^T+1T=∝rT+∝1−∝rT−1+∝1−∝2rT−2+,….

In Equation (4), the alpha parameter ∝ controls how much weight is given to recent versus past data. An ∝⟶0 favors older observations, while an ∝⟶1 gives more importance to recent data. The Holt–Winters algorithm extends the basic exponential smoothing approach by incorporating a seasonality component.

### 2.5. Forecasting With Count-Based Models (Poisson and Negative Binomial GLMs)

Count data, which are non-negative integers, are common in epidemiology, demography, and related fields. Such data often violate the assumptions of traditional continuous-valued forecasting models, leading to implausible negative predictions or failing to account for the variance structure inherent in counts. To address these issues, GLMs designed explicitly for count outcomes are widely used.

The Poisson regression model is the simplest and most commonly applied GLM for count data. It assumes that the mean and variance of the response are equal (equidispersion). Under the log link function, the expected value of the count at time.

*t* is expressed as

(5) $$\begin{array}{c} Ε\left[y_{t}\right]=\exp\left(\beta_{0}+\beta_{1}t\right), \end{array}$$

where *y*_*t*_ denotes the count at time *t*, and the parameters *β*_0_ and *β*_1_ capture the baseline and temporal trend, respectively. This specification ensures non-negative forecasts and provides interpretable coefficients in terms of multiplicative effects on the outcome [29, 30]. However, real-world count data frequently exhibit overdispersion, where the variance exceeds the mean. In such cases, the negative binomial regression model offers a more appropriate alternative. This model introduces an additional dispersion parameter, allowing for greater flexibility by relaxing the equidispersion assumption of the Poisson model [31]. As a result, the negative binomial GLM tends to provide more robust inference and more realistic uncertainty estimates when variability in the data is high.

### 2.6. Forecasting With the Stacking Model

**Figure 3:**
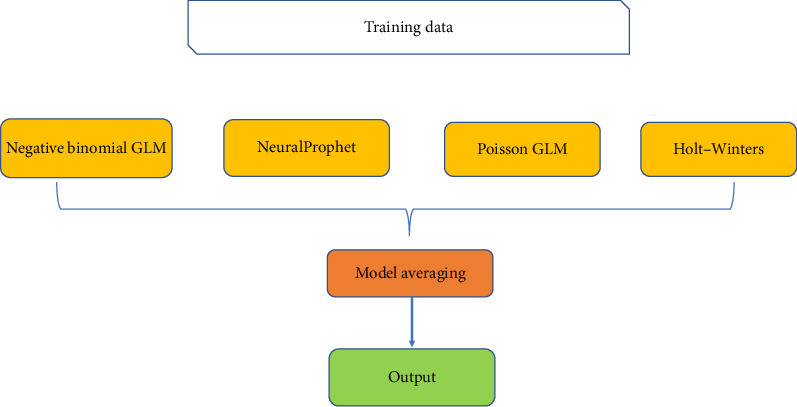
The framework used for building the stacking model.

To achieve a well-rounded performance, we combined the outputs of the MA, Holt–Winters exponential smoothing, NeuralProphet, and count-based GLMs (Poisson and negative binomial) into a stacking framework. Stacking models can be constructed using various approaches, including simple averaging, weighted averaging, or utilizing a meta-learner trained on the predictions of individual models. In this study, we employed simple model averaging to enhance predictive accuracy while mitigating the bias of any single method. The architecture of the stacking model is illustrated in Figure 3.

To evaluate the difference between observed and predicted values (error) for the models used in this study, the accuracy of each model was assessed using metrics including RMSE, mean squared error (MSE), and MAE.

## 3. Result and Discussion

### 3.1. Forecast Results' Analysis

**Figure 4:**
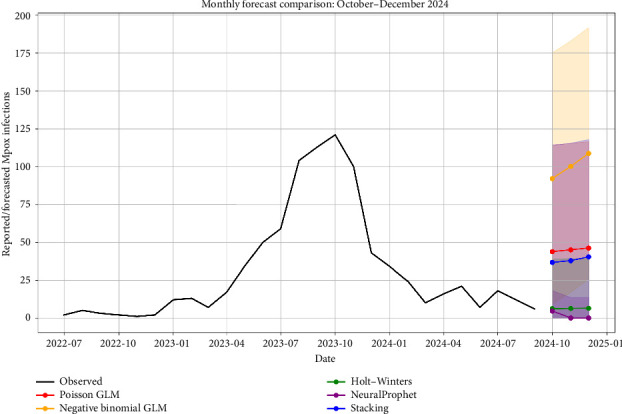
Observed monthly mpox cases alongside model-based forecasts (Poisson GLM, Negative binomial GLM, Holt–Winters, NeuralProphet, and Stacked ensemble) for October–December 2024, with 95% uncertainty intervals.

This section compares forecasts from the Poisson GLM, negative binomial GLM, Holt–Winters, NeuralProphet, and a stacked ensemble.  Figure 4 plots observed mpox cases alongside model-based projections, and Tables 2 and 3 report the forecasted values along with 95% prediction intervals and the out-of-sample evaluation metrics obtained via rolling origin cross-validation. Calibration assessed via rolling-origin cross-validation showed that Holt–Winters achieved 71.43% empirical coverage at the 95% level, NeuralProphet 35.71%, and the stacked ensemble 78.57%, indicating that the ensemble provided the most reliable uncertainty quantification in this dataset.

Holt–Winters produced stable forecasts of about six cases per month across October–December 2024 (Table 3) and achieved the lowest RMSE among single models (19.68), with 95% interval coverage of 71.43% (Table 2). The negative binomial GLM achieved the lowest MAE (4.04) but had a substantially higher RMSE (38.03) and lower coverage (64.29%), indicating larger occasional errors and less reliable intervals, despite a strong average absolute fit. The Poisson GLM exhibited a similar pattern to the negative binomial model, with moderate point errors (MAE: 7.31) but high RMSE (38.03), and a comparable coverage profile (64.29% at 95%).

NeuralProphet forecasted near-zero counts over the horizon (0–0–0), reflecting limited signal extraction in a short, low-incidence series. Its MAE (5.97) and RMSE (23.94) were competitive in terms of average magnitude, but interval calibration was weak (95% coverage: 35.71%), which limits reliability for uncertainty-aware interpretation. By contrast, the stacked ensemble delivered the most reliable uncertainty quantification, with the highest empirical coverage (78.57% at 95% and 71.43% at 80%) and an RMSE of 21.26. Its MAE (22.90) was higher than that of the single models, consistent with an approach that prioritizes calibrated intervals over point accuracy.

Overall, Holt–Winters is the strongest single-model option by RMSE with respectable calibration, while the ensemble provides the most trustworthy uncertainty characterization. In settings where interval reliability is crucial for situational awareness and contingency planning, the ensemble is preferable; when a single-point forecast is needed, Holt–Winters offers the best balance of stability and accuracy for this dataset. Prediction interval lower bounds in Table 3 are truncated at zero to respect the non-negativity of reported case counts, which appropriately concentrates uncertainty on the upper side under low-incidence conditions. GLM specifications with a log link ensure non-negative expected counts and accommodate overdispersion via the negative binomial distribution, making them well-suited to low-incidence surveillance series. While we included Holt–Winters and NeuralProphet as benchmarks, future work could evaluate state-space count models to capture latent dynamics. Beyond simple averaging, median- and quantile-based combinations, as well as quantile-regression stacking, could further improve interval calibration. We plan to evaluate these approaches as additional data become available.

### 3.2. Implications for Policymakers

The consolidated forecasts reveal meaningful differences in how models project mpox case counts in Thailand for late 2024. Count-based models, such as Poisson and negative binomial GLMs, tend to overestimate future cases, reflecting their sensitivity to past outbreak peaks. By contrast, Holt–Winters produced stable but conservative forecasts, while the stacking ensemble integrated across methods and yielded balanced predictions with more reliable uncertainty bounds. These divergences underscore the importance of interpreting forecasts as exploratory tools rather than definitive predictions, particularly given the limited data and short time horizon. These short-horizon forecasts are exploratory decision-support inputs and should be interpreted alongside real-time surveillance, operational constraints, and expert judgment as they are not prescriptive recommendations.

For policymakers, the primary value of these forecasts lies in clarifying potential scenarios rather than absolute numbers. Higher GLM-based forecasts emphasize preparedness for worst-case outcomes, while the more stable Holt–Winters results suggest the possibility of sustained low incidence if existing control measures remain effective. The stacking ensemble, by balancing across approaches, offers a pragmatic middle ground for short-term situational awareness.

Policy responses should therefore remain flexible and adaptive. Seasonal travel surges, particularly during Thailand's peak tourism months (November–February), can amplify transmission risk irrespective of baseline forecasts. While our analysis does not prescribe specific actions, agencies have considered options such as enhanced screening at ports of entry, public awareness campaigns, and contingency planning for rapid case escalation. We cite these solely as examples from other settings (e.g., the U.S. CDC emphasized targeted vaccination, proactive communication, and enhanced surveillance during its mpox response [32]); assessing their appropriateness for Thailand lies outside the scope of this study. These strategies align with global best practices; for example, the U.S. Centers for Disease Control and Prevention (CDC) emphasized targeted vaccination, proactive communication, and enhanced surveillance during its mpox response [32].

Ultimately, forecasts should be integrated with real-time surveillance rather than treated as standalone projections. Iteratively updating models with new case data will enhance their relevance, allowing Thailand's DDC to strengthen adaptive, evidence-based interventions under both low- and high-incidence scenarios.

## 4. Conclusion, Limitations, and Future Work

This study employed a combination of statistical and machine learning models, including Poisson GLM, negative binomial GLM, Holt–Winters, NeuralProphet, and a stacked ensemble, to forecast monthly mpox cases in Thailand from October to December 2024. Among the models, Holt–Winters provided the most stable single-model forecasts, while the stacking ensemble delivered the most reliable uncertainty calibration, making it particularly suitable for short-term situational awareness. These results underscore the importance of employing multiple forecasting approaches and ensembles to strike a balance between accuracy and uncertainty when working with limited datasets.

However, several limitations must be acknowledged. The dataset spanned only July 2022 to September 2024, restricting the ability of models to capture longer-term epidemic dynamics. The short 3-month forecast horizon further emphasizes that forecasts should be viewed as exploratory rather than definitive. The analysis was conducted at the native monthly frequency of Thailand's DDC reporting, avoiding artificial autocorrelation from daily interpolation but limiting granularity. Moreover, some models, particularly the Poisson and negative binomial GLMs, exhibited poor calibration and a tendency to overpredict or underestimate the uncertainty surrounding count-based forecasts in small samples. While reproducibility has been strengthened through documentation of parameter settings, preprocessing steps, and software versions, broader external validation will require continuous updates as new case data emerge. We did not evaluate multiplicative ETS or state-space count models (e.g., Poisson/NB state-space). Furthermore, the ensemble used simple averaging rather than median/quantile combinations or quantile-regression stacking. In addition, the prediction intervals were derived from residual variability and truncated at 0 (which can affect nominal coverage under low incidence). Lastly, no exogenous covariates (e.g., mobility or international travel flows) were included in the GLMs, Holt–Winters, NeuralProphet, or the stacked ensemble.

Future work should focus on extending the dataset with updated surveillance records, testing more advanced ensemble strategies (e.g., quantile regression stacking) to improve interval calibration, and incorporating exogenous predictors such as seasonal travel patterns or mobility data. Linking forecasts with real-time surveillance systems could enhance responsiveness and provide policymakers with adaptive tools to plan interventions. By treating forecasts as decision-support inputs that complement, rather than replace, epidemiological judgment, Thailand's public health authorities can better prepare for both low- and high-incidence scenarios in the evolving mpox outbreak.

## Figures and Tables

**Table 1:** Description of the mpox dataset from DDC Thailand.

s/n	Property	Value
1	Description	Time-series data
2	[Min, Max]	[0, 121]
3	Characteristics	No strong trend
4	Behavior	Nonstationary
5	Start month	July 2022
6	End month	September 2024
7	Outliers, missing	None

**Table 2:** Out-of-sample MAE, RMSE, MAPE, and empirical coverage (95%, 80%) computed via rolling-origin cross-validation with a minimum 12-month training window.

Models	MAE	RMSE	MAPE	Coverage @95 (%)	Coverage@80 (%)
Poisson GLM	7.31	38.03	113.20	64.29	57.14
Negative binomial GLM	4.04	38.03	113.20	64.29	57.14
Holt–Winters	6.54	19.68	63.52	71.43	64.29
NeuralProphet	5.97	23.94	74.16	35.71	14.30
Stacking	22.90	21.26	63.73	78.57	71.43

**Table 3:** Forecasted monthly mpox cases for October–December 2024 with 95% prediction intervals.

Month	Model	Forecasted case	Lower 95%	Upper 95%
October 2024	Poisson GLM	44	0	114
	Negative binomial GLM	92	9	175
	NeuralProphet	0	0	11
	Stacking	36	0	114
	Holt–Winters	6	0	39

November 2024	Poisson GLM	45	0	115
	Negative binomial GLM	100	17	183
	NeuralProphet	0	0	11
	Stacking	38	0	116
	Holt–Winters	6	0	39

December 2024	Poisson GLM	46	0	117
	Negative binomial GLM	109	26	192
	NeuralProphet	0	0	11
	Stacking	40	0	119
	Holt–Winters	6	0	39

*Note:* Lower bounds are truncated at 0 to reflect non-negative counts.

## Data Availability

The dataset supporting this study's findings is provided in Supporting File (Table S1), which contains monthly mpox case counts from July 2022 to September 2024 reported by the Department of Disease Control (DDC), Ministry of Public Health, Thailand.

## Appendix

The computational environment used was Python 3.11, with the following libraries: Pandas 2.2.2, NumPy 1.26.4, Matplotlib 3.9.2, StatsModels 0.14.2, NeuralProphet 0.6.2, and scikit-learn 1.5.1. For stochastic models (e.g., NeuralProphet), we fixed the random seed at 42 to ensure replicability. The full forecasting script is available on GitHub https://github.com/Ses4short/Mpox/blob/main/Thailand.

## Supporting Information

Supporting File: Table S1: Monthly Trend of Reported Mpox Infections in Thailand from July 2022 to September 2024.

## References

[1] Nii‐Trebi N. I., Mughogho T. S., Abdulai A. (2023). Dynamics of Viral Disease Outbreaks: A Hundred Years (1918/19–2019/20) in Retrospect‐Loses, Lessons and Emerging Issues. *Reviews in Medical Virology*.

[2] Chaudhary R., Rohilla M., Chauhan S. (2023). The Pandemic’s Unseen Wounds: COVID-19’s Profound Effects on Mental Health. *Annals of Medicine and Surgery*.

[3] Isasi F., Naylor M. D., Skorton D., Grabowski D., Hernandez S., Montgomery Rice V. (2021). Patients, Families, and Communities COVID-19 Impact Assessment: Lessons Learned and Compelling Needs. *NAM Perspectives*.

[4] (2024). *WHO Director-General Declares Mpox Outbreak a Public Health Emergency of International Concern*.

[5] Cann J. A., Jahrling P. B., Hensley L. E., Wahl-Jensen V. (2013). Comparative Pathology of Smallpox and Monkeypox in Man and Macaques. *Journal of Comparative Pathology*.

[6] (2024). *Epidemiological Update: Mpox Due to Monkeypox Virus Clade I*.

[7] (2024). *FACT SHEET: United States Response to the Clade I Mpox Outbreak*.

[8] Srithammavong D., Srihawan C., Kittiyaowamarn R., Suphanchaimat R., Yingyong T., Bunyakitikorn W. (2025). Case Report of Clade Ib Monkeypox Virus Infection Linked to Travel to Democratic Republic of the Congo, Thailand, 2024. *Emerging Infectious Diseases*.

[9] Yang H. L. (2024). *Commentary: Global Mpox Emergency–The World Must Show It Learnt Its Lessons From COVID-19*.

[10] Walderich A. (2024). *Tourism Industry in Thailand-Statistics & Facts*.

[11] Riyapan P., Shuaib S. E., Intarasit A. (2021). A Mathematical Model of COVID-19 Pandemic: A Case Study of Bangkok, Thailand. *Computational and Mathematical Methods in Medicine*.

[12] (2023). *Managing Epidemics: Key Facts About Major Deadly Diseases*.

[13] Hartley D. M., Nelson N. P., Arthur R. R. (2013). An Overview of Internet Biosurveillance. *Clinical Microbiology and Infection*.

[14] Dixon S., Keshavamurthy R., Farber D. H., Stevens A., Pazdernik K. T., Charles L. E. (2022). A Comparison of Infectious Disease Forecasting Methods Across Locations, Diseases, and Time. *Pathogens*.

[15] Mugglin A. S., Cressie N., Gemmell I. (2002). Hierarchical Statistical Modelling of Influenza Epidemic Dynamics in Space and Time. *Statistics in Medicine*.

[16] Overton C. E., Stage H. B., Ahmad S. (2020). Using Statistics and Mathematical Modelling to Understand Infectious Disease Outbreaks: COVID-19 as an Example. *Infectious Disease Modelling*.

[17] Huppert A., Katriel G. (2013). Mathematical Modelling and Prediction in Infectious Disease Epidemiology. *Clinical Microbiology and Infection*.

[18] Kremer C., Torneri A., Boesmans S. (2021). Quantifying Super Spreading for COVID-19 Using Poisson Mixture Distributions. *Scientific Reports*.

[19] Zhao S., Shen M., Musa S. S. (2021). Inferencing Superspreading Potential Using Zero-Truncated Negative Binomial Model: Exemplification With COVID-19. *BMC Medical Research Methodology*.

[20] Ahundjanov B. B., Akhundjanov S. B., Okhunjanov B. B. (2022). Power Law in COVID-19 Cases in China. *Journal of the Royal Statistical Society-Series A: Statistics in Society*.

[21] Department of Disease Control *Monkeypox (Mpox) Situation Report*.

[22] Williams B., Lacy P. S., Yan P., Hwee C. N., Liang C., Ting C. M. (2011). Development and Validation of a Novel Method to Derive Central Aortic Systolic Pressure From the Radial Pressure Waveform Using an N-Point Moving Average Method. *Journal of the American College of Cardiology*.

[23] Taylor S. J., Letham B. (2018). Forecasting at Scale. *The American Statistician*.

[24] Taylor S. J., Letham B. *Prophet: Automatic Forecasting Procedure, R Package Version 0.5*.

[25] Triebe O., Laptev N., Rajagopal R. (2019). *Ar-net: A Simple Auto-Regressive Neural Network for Time-Series*.

[26] Brown R. G. (1959). *Statistical Forecasting for Inventory Control*.

[27] Holt C. C. (2004). Forecasting Seasonals and Trends by Exponentially Weighted Moving Averages. *International Journal of Forecasting*.

[28] Winters P. R. (1960). Forecasting Sales by Exponentially Weighted Moving Averages. *Management Science*.

[29] Cameron A. C., Trivedi P. K. (2013). *Regression Analysis of Count Data*.

[30] Hilbe J. M. (2011). *Negative Binomial Regression*.

[31] McCullagh P. (2019). *Generalized Linear Models*.

[32] Kava C. M., Rohraff D. M., Wallace B. (2022). Epidemiologic Features of the Monkeypox Outbreak and the Public Health Response—United States, May 17–October 6, 2022. *MMWR, Morbidity and Mortality Weekly Report*.

