# Supplementary material for: Forecasting the Monkeypox Outbreak Using Limited Data: A Case Study of Thailand
Source: Interdiscip Perspect Infect Dis. 2025 Nov 24;2025:7199833. doi: 10.1155/ipid/7199833 (PMC12668862; doi:10.1155/ipid/7199833)
Supplement: Supporting Information — Additional supporting information can be found online in the Supporting Information section. [file 7199833.f1.docx]

The data used in this study was publicly available from the Department of Disease Control, Ministry of Public Health, Thailand [https://ddc.moph.go.th/monkeypox/dashboard.php] Last accessed on 23^rd^ September, 2024.

Table S1 Monthly Trend of Reported Mpox Infections in Thailand from July 2022 to September 2024

| Year | Month | No of Reported infections | |
| --- | --- | --- | --- |
| 2022 | July | 2 |  |
| 2022 | August | 5 |  |
| 2022 | September | 3 |  |
| 2022 | October | 2 |  |
| 2022 | November | 1 |  |
| 2022 | December | 2 |  |
| 2023 | January | 12 |  |
| 2023 | February | 13 |  |
| 2023 | March | 7 |  |
| 2023 | April | 17 |  |
| 2023 | May | 34 |  |
| 2023 | June | 50 |  |
| 2023 | July | 59 |  |
| 2023 | August | 104 |  |
| 2023 | September | 113 |  |
| 2023 | October | 121 |  |
| 2023 | November | 100 |  |
| 2023 | December | 43 |  |
| 2024 | January | 34 |  |
| 2024 | February | 24 |  |
| 2024 | March | 10 |  |
| 2024 | April | 16 |  |
| 2024 | May | 21 |  |
| 2024 | June | 7 |  |
| 2024 | July | 18 |  |
| 2024 | August | 12 |  |
| 2024 | September | 6 |  |
